# USP44 Stabilizes MAOB via Deubiquitination to Inhibit Cisplatin Resistance in Lung Adenocarcinoma

**DOI:** 10.1155/ijog/7433804

**Published:** 2026-05-22

**Authors:** Zhi Liang, Hui Zhang, Jinhua Jiang, Chuanchuan Li, Feng Yuan

**Affiliations:** ^1^ Graduate School, Zhejiang Chinese Medical University, Hangzhou, China, zcmu.edu.cn; ^2^ Department of Thoracic Surgery, Linping Campus, The Second Affiliated Hospital of Zhejiang University School of Medicine, Hangzhou, China, z2hospital.com; ^3^ Nursing Department of Gudang Sub-district Community Health Service Center, Xihu District, Hangzhou, China; ^4^ Department of Cardiothoracic and Vascular Surgery, Kecheng District People’s Hospital of Quzhou City, Quzhou, China; ^5^ Department of Thoracic Surgery, Zhejiang Hospital, Hangzhou, China, zjhospital.com.cn

**Keywords:** cisplatin, epithelial–mesenchymal transition, lung adenocarcinoma, monoamine oxidase B, ubiquitin-specific protease 44

## Abstract

**Background:**

Cisplatin (DDP), as a commonly used chemotherapeutic agent for the clinical treatment of lung adenocarcinoma (LUAD), serves a key function in suppressing tumor progression; however, the development of tumor cell resistance has restricted its clinical application.

**Methods:**

Differentially expressed genes (DEGs) between A549 and A549‐DDP cells in the GEO dataset (GSE157692) were analyzed, and DDP resistance–related targets were downloaded from GeneCards; candidate genes were obtained via their intersection. Kyoto Encyclopedia of Genes and Genomes (KEGG) and Gene Ontology (GO) analyses were conducted on candidate genes. Subsequently, key gene expression was analyzed via GEPIA and TCGA databases and verified by Western blot. Cell proliferation, apoptosis, migration, and invasion were detected by CCK‐8, colony formation, flow cytometry, and Transwell assays. The interaction between MAOB and USP44 was predicted by UbiBrowser, and their regulatory relationship was validated using coimmunoprecipitation (Co‐IP) and cycloheximide (CHX) chase assays.

**Results:**

Monoamine oxidase B (MAOB) was identified as a core target. MAOB showed reduced expression in DDP‐resistant LUAD cells and tumor tissues, with low levels linked to worse prognosis. MAOB overexpression enhanced DDP sensitivity, repressed cell proliferation/migration/invasion, promoted apoptosis, and reversed epithelial–mesenchymal transition (EMT). Ubiquitin‐specific protease 44 (USP44) was downregulated in DDP‐resistant LUAD models and stabilized MAOB via deubiquitination. MAOB knockdown reversed USP44 overexpression’s effects on DDP resistance and malignant phenotypes.

**Conclusion:**

USP44 promoted MAOB protein stability via deubiquitination, and the USP44‐MAOB axis inhibited DDP resistance and malignant phenotypes of LUAD cells. This axis is thus a potential therapeutic target for improving DDP resistance in LUAD, providing a new direction for clinical intervention.

## 1. Introduction

Lung adenocarcinoma (LUAD) stands among the deadliest malignancies worldwide, noted for challenges in early detection, high rates of recurrence and metastasis, and poor clinical outcomes [1]. Currently, cisplatin (DDP)‐based chemotherapy regimens remain the first‐line treatment option for advanced LUAD [2]. However, the widespread development of DDP resistance severely limits treatment efficacy; most patients experience disease progression due to resistance during treatment cycles, ultimately resulting in an extremely poor prognosis [3]. Although existing studies have confirmed that DDP resistance involves multiple aspects, including increased drug efflux, enhanced DNA damage repair, inhibited cell apoptosis, and tumor microenvironment remodeling [4, 5], currently identified regulatory targets still fail to fully explain the resistance phenomenon and cannot meet the needs of clinical intervention. Therefore, identifying new key molecules and elucidating their regulatory mechanisms has become a core direction for overcoming the bottleneck of DDP resistance in LUAD.

Monoamine oxidase B (MAOB), a core metabolic enzyme localized on the mitochondrial membrane, has drawn growing interest in oncology research in recent years [6] and exhibits significant tumor specificity: In gliomas, MAOB is highly expressed, and its level is directly associated with poor patient prognosis [7]; conversely in prostate cancer studies, high MAOB levels are significantly correlated with favorable patient prognosis [8]. These findings collectively identify MAOB as a potential regulator of tumor progression. Yet a critical gap exists in LUAD: MAOB’s specific role in DDP resistance is unknown, and no systematic studies explore upstream molecules that stabilize MAOB, regulate its function, or synergize to affect LUAD cells’ drug‐resistant phenotype. This gap limits both a full understanding of MAOB’s biological roles and its clinical value as a LUAD resistance intervention target. Based on the preliminary results, MAOB was screened as the most significantly downregulated gene in DDP‐resistant A549‐DDP cells, but its specific functions and regulatory mechanisms in DDP resistance remain unclear. Thus, in the present study, we aimed to further explore the roles of MAOB in DDP resistance in LUAD and clarify its upstream regulatory network.

By integrating bioinformatics analyses, in vitro cellular experiments, and functional validation assays, this study systematically investigates the biological function of MAOB in DDP‐resistant LUAD cells and its underlying regulatory mechanisms. Beyond enriching the molecular network of LUAD resistance mechanisms, this work is aimed at supplying novel theoretical foundations and potential molecular candidates for the clinical development of targeted therapies to combat DDP resistance in LUAD.

## 2. Materials and Methods

### 2.1. Cell Culture

The parental LUAD cell lines (A549 and PC9) and their corresponding DDP‐resistant derivatives (A549‐DDP and PC9‐DDP) were obtained from Procell Biotechnology Co., Ltd. (Wuhan, China). Cells were cultured in a humidified incubator at 37°C with 5% CO_2_. For routine maintenance to preserve their phenotypes, A549 cells were cultured in Ham’s F‐12K medium (Procell, Catalog No. PM150910) supplemented with 10% fetal bovine serum (FBS; Procell, Catalog No. 164210) and 1% penicillin–streptomycin (P/S; Procell, Catalog No. PB180120). A549‐DDP cells, to maintain DDP resistance, were maintained in the same Ham’s F‐12K medium formulation with the addition of 1 *μ*g/mL DDP. Similarly, PC9 cells were cultured in RPMI‐1640 medium (Procell, Catalog No. PM150110) containing 10% FBS and 1% P/S, while PC9‐DDP cells were maintained in the same RPMI‐1640 medium as PC9 with 1 *μ*g/mL DDP added to preserve resistance.

### 2.2. Screening of DDP Resistance Targets

Profiles of gene expression in A549 and A549/DDP cells were obtained from the Gene Expression Omnibus (GEO) dataset GSE157692 (https://www.ncbi.nlm.nih.gov/geo/query/acc.cgi?acc=GSE157692). Differentially expressed genes (DEGs) were screened using the criteria of adjusted *p* value (adj*p*) < 0.05 and ∣log fold change (logFC) | ≥2. Meanwhile, targets associated with DDP resistance were downloaded from the GeneCards database (https://www.genecards.org/) by querying the term “Cisplatin resistance.” Subsequently, the intersection between the DEGs obtained from GSE157692 and the DDP resistance–related targets from GeneCards was determined to identify candidate genes implicated in DDP resistance.

### 2.3. Gene Ontology (GO) and Kyoto Encyclopedia of Genes and Genomes (KEGG) Enrichment Analyses

The intersection genes obtained from the above screening were subjected to GO and KEGG pathway enrichment analyses using the SangeBox online platform (https://sangebox.com/). Briefly, the list of intersection genes was uploaded into the platform, and enrichment analyses were performed with default parameters, focusing on GO annotations, including biological process (BP), cellular component (CC), and molecular function (MF), and KEGG signaling pathways. Enrichment results were filtered using the threshold of *p* value < 0.05. For both GO terms and KEGG pathways, the filtered results were sorted in ascending order based on *p* values. The Top 10 most significantly enriched items were selected, and bubble plots were generated using the built‐in plotting function of SangeBox to present these results.

### 2.4. Bioinformatic Analyses

MAOB and USP44 expression in LUAD tumor tissues versus normal tissues, as well as the prognostic significance of MAOB expression for LUAD patients, were analyzed using the GEPIA (http://gepia.cancer-pku.cn/) and TCGA database (https://portal.gdc.cancer.gov). Meanwhile, the online tool UbiBrowser (http://ubibrowser.bio-it.cn/) was employed to predict the interaction between MAOB and USP44.

### 2.5. Cell Transfection

Cell transfection was performed using Lipofectamine 2000 (Invitrogen) following the manufacturer’s protocol. Cells were seeded in culture plates 24 h prior to transfection to reach 80% confluence at the time of transfection. For overexpression experiments, MAOB and USP44 overexpression plasmids (designated as MAOB and USP44) were constructed using the pcDNA3.1 vector (GenePharma, Shanghai, China), with the empty pcDNA3.1 vector (designated as pcDNA) serving as the negative control. For gene silencing, MAOB‐specific small interfering RNA (siMAOB) and nontargeting negative control siRNA (siNC) were purchased from GenePharma (Shanghai, China).

### 2.6. Western Blot

Cell lysates were prepared in RIPA buffer with protease/phosphatase inhibitors. Protein samples were resolved by 10% SDS‐PAGE and wet‐transferred to PVDF membranes (Millipore, Billerica, Massachusetts, United States). Membranes were blocked in 5% skim milk for 2 h at 25°C and then incubated with primary antibodies overnight at 4°C. After washing, membranes were incubated with HRP‐conjugated secondary antibodies for 1.5 h, rewashed, and visualized using Clarity Western ECL Substrate Kit (Bio‐Rad, Shanghai, China) with a gel imager for grayscale analysis. The primary antibodies included anti‐MAOB (1:1000, Abcam, Cambridge, UK, Catalog No. ab133270), anti‐E‐cadherin (1:1000, Abcam, Catalog No. ab219332), anti‐N‐cadherin (1:1000, Abcam, Catalog No. ab207608), anti‐vimentin (1:1000, Abcam, Catalog No. ab16700), anti‐USP44 (1:500, Proteintech, Wuhan, China, Catalog No. 15521‐1‐AP), and anti‐*β*‐actin (1:10000, Proteintech, Catalog No. 66009‐1‐Ig). Corresponding secondary antibodies were Goat Anti‐Rabbit/Mouse IgG H&L (1:5000, Abcam, Catalog No. ab6721/ab6789).

### 2.7. Cell Viability Detection

Cell viability was evaluated using the Cell Counting Kit‐8 (CCK‐8) (Solarbio, Beijing, China, Catalog No. CA1210). Cells were seeded into 96‐well plates at ~5 × 10^3^ cells/well. Then, 10 *μ*L CCK‐8 reagent was added. Absorbance at 450 nm was measured with a microplate reader, and viability was calculated as a percentage relative to the untreated controls. The half‐maximal inhibitory concentration (IC_50_) values were analyzed using GraphPad Prism software by fitting concentration‐response curves.

### 2.8. Cell Clonogenic Capacity Assessment

Cells were seeded into 6‐well plates (500 cells/well) to ensure the formation of discrete colonies. After seeding, cells were cultured in complete medium at 37°C with 5% CO_2_ for 14 days, with medium refreshed every 3–4 days. Upon visible colony formation, the medium was aspirated; colonies were fixed with 4% paraformaldehyde (Beyotime) and stained with 0.1% crystal violet (Beyotime). Colonies were imaged and counted. Colony formation rate was calculated as (colonies formed/seeded cells) × 100*%*.

### 2.9. Flow Cytometry

The Annexin V‐FITC/PI Apoptosis Detection Kit (Yeasen, Shanghai, China, Catalog No. 40302ES50) was employed to assess cell apoptosis. Briefly, cells were harvested and washed twice with precooled PBS. Thereafter, 1 × 10^5^ cells were collected and resuspended in 1× binding buffer. Subsequently, Annexin V‐FITC and PI Staining Solution were added, and the cell suspension was then incubated for 15 min in the dark. Next, 1× binding buffer was added, and the samples were analyzed using flow cytometry.

### 2.10. Transwell Assay

Cell migration and invasion were assessed using Transwell chambers (Corning, Tewksbury, Massachusetts, United States). For invasion, Matrigel matrix (Bedford, Massachusetts, United States) was diluted 1:8 in serum‐free medium; 50 *μ*L of the diluted Matrigel was added to the upper Transwell chamber and incubated at 37°C for 60 min. For migration, no Matrigel was applied. Cells were resuspended in serum‐free medium at 5 × 10^4^ cells/mL. Two hundred microliters of the cell suspension was added to the upper chamber, while 600 *μ*L of complete medium was added to the lower chamber. After incubation, nonmigrated/noninvaded cells were wiped away. Cells were fixed with 4% paraformaldehyde and stained with 0.1% crystal violet. Migrated/invaded cells were imaged via light microscopy.

### 2.11. Ubiquitination Assessment

Coimmunoprecipitation (Co‐IP) assays were performed to detect MAOB ubiquitination. Cells were lysed in ice‐cold IP lysis buffer supplemented with protease inhibitors and deubiquitinase (DUB) inhibitors. Cell lysates were centrifuged, and the supernatants were collected. For IP, the supernatants were incubated with an anti‐MAOB antibody (Abcam, Catalog No. ab133270) overnight at 4°C, followed by incubation with Protein A/G agarose beads (Thermo Fisher Scientific, Waltham, Massachusetts, United States) for an additional 2 h. The immunoprecipitated proteins were eluted by boiling in SDS loading buffer and then subjected to Western blot using antibodies against ubiquitin (Ub), MAOB, USP44, and *β*‐actin. The input lysates (a portion of the cell lysates before IP) were also analyzed by Western blot to verify protein expression levels.

### 2.12. Cycloheximide (CHX) Chase Assay

To evaluate the stability of the MAOB protein, CHX chase assays were performed. Briefly, cells were treated with 50 *μ*g/mL CHX (Sigma‐Aldrich, St. Louis, Missouri, United States) and then harvested at 0, 4, and 8 h. Western blot was used to detect MAOB expression. The residual MAOB protein levels at each time point were analyzed to assess protein stability.

### 2.13. Statistical Analysis

All data were presented as the mean ± standard deviation (SD) from at least three independent biological replicates (*n* ≥ 3, where *n* represents the number of independent replicates per group). For comparisons between two groups, an unpaired *t*‐test was utilized. For multiple‐group comparisons, one‐way analysis of variance (one‐way ANOVA) followed by Tukey’s post hoc test was performed, with a value of *p* < 0.05 considered statistically significant.

## 3. Results

### 3.1. Bioinformatic Identification of Targets Associated With DDP Resistance in LUAD

**Figure 1:**
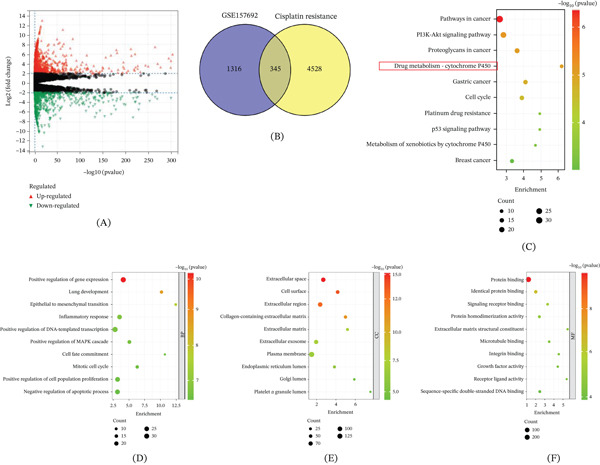
Bioinformatic analysis of DDP resistance–related genes in LUAD. (A) Volcano plot depicting DEGs in DDP‐resistant cells. (B) Venn diagram illustrating the intersection between genes from the GSE157692 dataset and DDP resistance–related targets, identifying 345 overlapping core genes. (C) KEGG pathway enrichment analysis of the core genes. (D–F) GO enrichment analysis for BP, CC, and MF.

To elucidate the molecular mechanisms underlying DDP resistance in LUAD, a series of bioinformatic analyses were performed. First, a volcano plot was generated to identify DEGs in DDP‐resistant cells (Figure 1A); numerous genes were found to be significantly upregulated or downregulated, indicating widespread transcriptional alterations associated with DDP resistance. Core genes in DDP resistance were identified by intersecting GSE157692‐derived genes with DDP resistance–related targets from GeneCards via Venn analysis. As shown in Figure 1B, 345 overlapping genes were identified, which were regarded as pivotal candidates for investigating DDP resistance mechanisms. In KEGG pathway analysis (Figure 1C), pathways such as “drug metabolism‐cytochrome P450” and “platinum drug resistance” were significantly enriched, highlighting the involvement of drug metabolic processes and resistance‐related signaling in DDP resistance. For BPs (Figure 1D), DEGs were enriched in terms including “positive regulation of gene expression” and “epithelial to mesenchymal transition.” For CCs (Figure 1E), enrichment was observed in “extracellular space” and “cell surface,” indicating potential involvement in intercellular and cell‐matrix interactions. For MFs (Figure 1F), DEGs were predominantly enriched in “protein binding.” Together, these analyses systematically identified DEGs, key pathways, and functional modules associated with DDP resistance in LUAD.

### 3.2. Expression Analysis of MAOB Reveals Its Downregulation in DDP‐Resistant LUAD and Prognostic Significance

**Figure 2:**
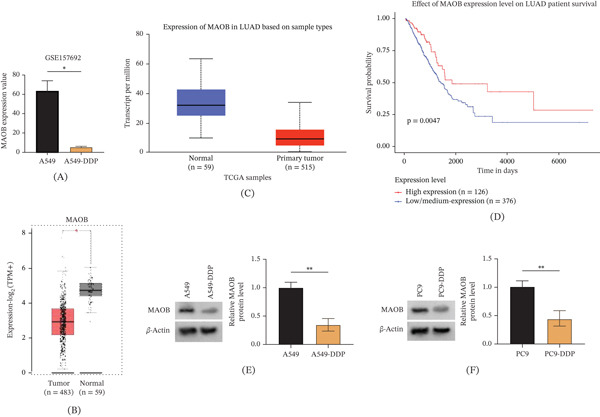
Expression and prognostic analysis of MAOB in LUAD and DDP‐resistant cell lines. (A) MAOB expression in A549 and A549‐DDP cells was analyzed using the GSE157692 dataset. (B) MAOB expression in LUAD (*n* = 483) and normal tissues (*n* = 59) was assessed via GEPIA. (C) Transcript levels of MAOB in normal (*n* = 59) and primary LUAD tumor (*n* = 515) samples from TCGA were compared. (D) Kaplan–Meier survival analysis of LUAD patients stratified by MAOB expression level (*p* = 0.0047). (E, F) MAOB protein levels in A549/A549‐DDP and PC9/PC9‐DDP cells were detected by Western blot.  ^∗^ *p* < 0.05;  ^∗∗^ *p* < 0.01.

To elucidate the role of genes enriched in the KEGG pathways, the expression pattern of MAOB was specifically investigated. In A549‐DDP cells, MAOB expression was found to be significantly downregulated, as demonstrated by the analysis of the GSE157692 dataset (Figure 2A). Subsequently, to identify the clinical expression profile of MAOB in LUAD, analyses were performed using the GEPIA and TCGA databases. It was observed that MAOB exhibited low expression in LUAD tissues (Figure 2B,C). Furthermore, survival analysis uncovered that low MAOB expression in LUAD patients was linked to poorer prognosis compared with high expression (Figure 2D, *p* = 0.0047), suggesting that MAOB expression level might serve as a prognostic biomarker. Notably, MAOB mRNA expression was significantly downregulated in DDP‐resistant LUAD tissues compared with DDP‐sensitive tissues (Figure S1, *p* < 0.001), confirming its association with clinical DDP resistance. Additionally, MAOB protein levels were remarkably downregulated in A549‐DDP and PC9‐DDP cell lines (Figure 2E,F). Collectively, MAOB was downregulated in both DDP‐resistant LUAD cell lines and tumor tissues, and its low expression was associated with an unfavorable prognosis in patients.

### 3.3. MAOB Overexpression Enhances DDP Sensitivity in DDP‐Resistant LUAD Cells Through Proliferation Inhibition and Apoptosis Promotion

**Figure 3:**
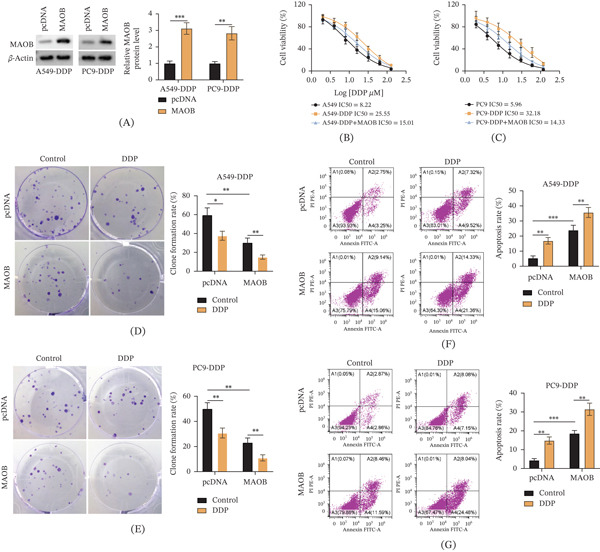
MAOB overexpression enhances DDP sensitivity in DDP‐resistant LUAD cells by inhibiting proliferation and promoting apoptosis. (A) MAOB overexpression efficiency was validated in A549‐DDP and PC9‐DDP cells via Western blot. (B, C) CCK‐8 assays were performed to determine DDP sensitivity in A549‐DDP and PC9‐DDP cells. (D–G) A549‐DDP and PC9‐DDP cells were transfected with pcDNA or MAOB‐overexpressing plasmid and divided into two subgroups: control (without DDP treatment) and DDP treatment. (D, E) Colony formation assays were conducted to assess cell proliferation in A549‐DDP and PC9‐DDP cells. (F, G) Flow cytometry analysis was used to detect cell apoptosis. Data are presented as mean ± SD from *n* ≥ 3 independent biological replicates.  ^∗^ *p* < 0.05;  ^∗∗^ *p* < 0.01.

Subsequently, a series of in vitro experiments were performed after establishing MAOB‐overexpressing models to explore MAOB’s functional role in DDP‐resistant LUAD cells. Western blot analysis (Figure 3A) confirmed the successful construction of the MAOB‐overexpressing cell models. Next, MAOB overexpression reduced the IC_50_ of DDP‐resistant A549‐DDP (from 25.55 to 15.01) and PC9‐DDP (from 32.18 to 14.33) cells, thereby partially reversing DDP resistance compared to their parental A549 (IC_50_ = 8.22) and PC9 (IC_50_ = 5.96) cells (Figure 3B,C). In addition, DDP treatment significantly inhibited colony formation in both the pcDNA group and the MAOB group; moreover, the colony formation rate of the MAOB group was lower than that of the pcDNA group under both control and DDP treatment (Figure 3D,E). Furthermore, DDP treatment significantly induced cell apoptosis, and overexpression of MAOB further enhanced the proapoptotic effect of DDP on cells (Figure 3F,G). In conclusion, these findings demonstrated that MAOB upregulation enhanced DDP sensitivity in DDP‐resistant LUAD cells.

### 3.4. MAOB Overexpression Suppresses Migration, Invasion, and Epithelial–Mesenchymal Transition (EMT) in DDP‐Resistant LUAD Cells

**Figure 4:**
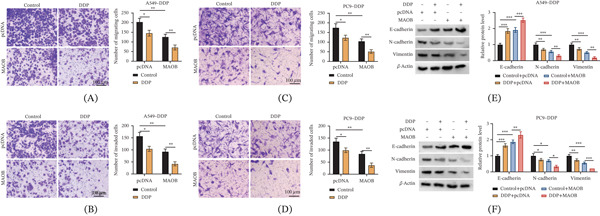
MAOB overexpression enhances DDP‐mediated inhibition of migration, invasion, and EMT in DDP‐resistant LUAD cells. Untreated or DDP‐treated A549‐DDP and PC9‐DDP cells were transfected with pcDNA or MAOB. (A–D) Transwell migration assay was conducted to assess cell migration and invasion. (E, F) Western blot analysis of EMT marker (E‐cadherin, N‐cadherin, and vimentin) expression levels. Data are presented as mean ± SD from *n* ≥ 3 independent biological replicates.  ^∗^ *p* < 0.05;  ^∗∗^ *p* < 0.01;  ^∗∗∗^ *p* < 0.001.

To further assess the effects of MAOB on the aggressive biological behaviors of DDP‐resistant LUAD cells, migration, invasion, and EMT were investigated. DDP treatment alone (pcDNA + DDP group) significantly inhibited cell migration and invasion. Notably, MAOB overexpression (MAOB + DDP group) further augmented DDP’s suppressive effect on migration and invasion (Figure 4A–D). For EMT regulation, a Western blot was conducted to detect key markers. In DDP‐treated cells, MAOB overexpression resulted in a more pronounced elevation in E‐cadherin and reduction in N‐cadherin and vimentin in comparison with the DDP + pcDNA group (Figure 4E,F). These findings demonstrated that DDP suppressed migration, invasion, and EMT in DDP‐resistant LUAD cells, and MAOB upregulation significantly enhanced these inhibitory effects.

### 3.5. USP44 Stabilizes MAOB via Deubiquitination in DDP‐Resistant LUAD Cells

**Figure 5:**
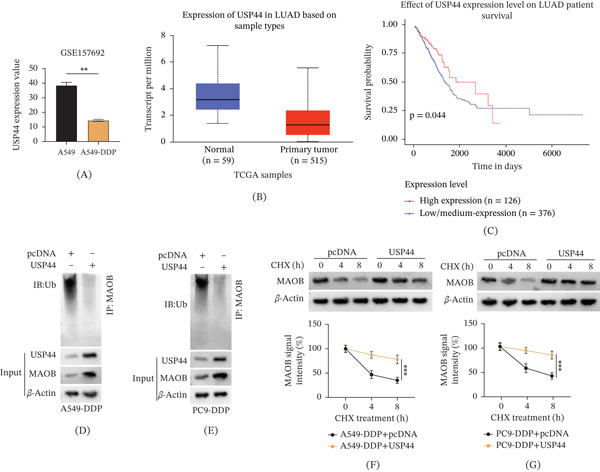
USP44 stabilizes MAOB via deubiquitination in DDP‐resistant LUAD cells. (A) USP44 expression in A549 and A549‐DDP cells was analyzed using the GSE157692 dataset. (B) Transcript levels of USP44 in normal (*n* = 59) and primary LUAD tumor (*n* = 515) samples from TCGA were compared. (C) Kaplan–Meier survival analysis of LUAD patients stratified by USP44 expression level (*p* = 0.044). (D, E) Co‐IP assays in A549‐DDP and PC9‐DDP cells showed that USP44 overexpression decreased MAOB ubiquitination. (F, G) CHX chase assay was employed to assess MAOB protein stability in A549‐DDP and PC9‐DDP cells transfected with pcDNA or USP44. Data are presented as mean ± SD from *n* ≥ 3 independent biological replicates.  ^∗∗^ *p* < 0.01;  ^∗∗∗^ *p* < 0.001.

Given that MAOB has been demonstrated to regulate DDP resistance in LUAD in previous analyses, the upstream regulatory mechanism governing MAOB was further explored. First, MAOB was identified as a potential substrate of USP44 through online UbiBrowser analysis, which provided a critical direction for investigating the molecular crosstalk between USP44 and MAOB. Subsequently, it was found that USP44 expression was downregulated in DDP‐resistant LUAD cells (Figure 5A). Further analysis of TCGA samples revealed that USP44 also exhibited low expression in primary LUAD tumor tissues relative to adjacent normal tissues (Figure 5B). Notably, LUAD patients characterized by low USP44 expression had poorer prognostic profiles (Figure 5C, *p* = 0.044). To verify the molecular interaction between USP44 and MAOB, Co‐IP assays were performed (Figure 5D,E). When USP44 was overexpressed, the ubiquitination level of MAOB was significantly reduced, indicating that USP44 could mediate the deubiquitination of MAOB. To further confirm the effect of USP44 on MAOB protein stability, CHX chase assays were conducted. USP44 overexpression markedly prolonged the half‐life of MAOB protein (Figure 5F,G), demonstrating that USP44 stabilized MAOB by inhibiting its Ub‐dependent degradation. Taken together, USP44 exhibited low expression levels in DDP‐resistant LUAD cells and interacted with MAOB to enhance its stability via deubiquitination.

### 3.6. USP44 Mediates DDP Resistance in LUAD Cells via Stabilizing MAOB

**Figure 6:**
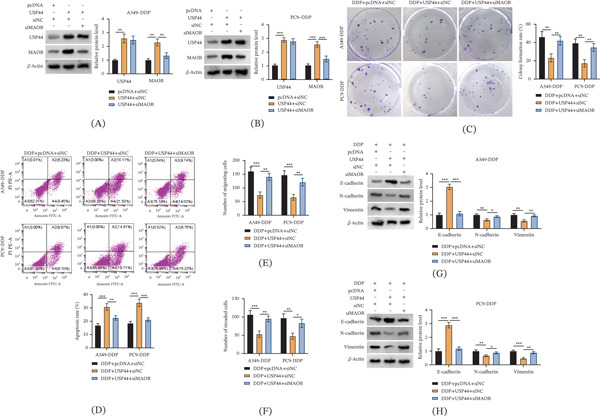
MAOB knockdown reverses the effects of USP44 overexpression on DDP resistance, proliferation, apoptosis, migration, invasion, and EMT in LUAD cells. A549‐DDP and PC9‐DDP cells were divided into three groups: pcDNA + siNC, USP44 + siNC, and USP44 + siMAOB. (A, B) Western blot was used to validate the efficiency of MAOB knockdown in USP44‐overexpressing A549‐DDP and PC9‐DDP cells. (C) Colony formation assay was used to assess cell proliferation. (D) Flow cytometry analysis of cell apoptosis. (E, F) Transwell assay was performed to evaluate cell migration and invasion. (G, H) Western blot analysis of E‐cadherin, N‐cadherin, and vimentin expression. Data are presented as mean ± SD from *n* ≥ 3 independent biological replicates.  ^∗^ *p* < 0.05;  ^∗∗^ *p* < 0.01;  ^∗∗∗^ *p* < 0.001.

To determine whether MAOB was the functional mediator of USP44, rescue experiments with MAOB knockdown were performed. First, the efficiency of MAOB knockdown in USP44‐overexpressing cells was validated by Western blot (Figure 6A,B). Moreover, it was confirmed that MAOB knockdown did not alter endogenous USP44 protein levels in DDP‐resistant cells, ruling out a reciprocal regulatory effect (Figure S2). USP44 overexpression increased MAOB protein levels, while cotransfection with siMAOB notably reduced MAOB expression, confirming the successful establishment of the rescue model. Additionally, USP44 overexpression inhibited colony formation and enhanced DDP‐induced apoptosis in both cell lines, yet these effects were reversed by MAOB knockdown (Figure 6C,D). For metastatic potential, USP44 upregulation inhibited the migratory and invasive capacities of DDP‐resistant LUAD cells, and these inhibitory effects were reversed by MAOB knockdown (Figure 6E,F). Moreover, USP44 overexpression upregulated E‐cadherin and downregulated N‐cadherin and vimentin (Figure 6G,H). Notably, MAOB silencing reversed these EMT‐related changes, suggesting that USP44‐mediated EMT regulation was MAOB‐dependent. Collectively, these data highlighted the pivotal role of the USP44‐MAOB axis in LUAD’s response to DDP and aggressive progression.

## 4. Discussion

LUAD is a highly lethal malignancy globally [9]. DDP‐based chemotherapy is a first‐line option for advanced cases, but primary or acquired DDP resistance severely compromises efficacy. Though progress has been made in exploring resistance mechanisms, complex molecular networks still hinder the development of reversal strategies. This study reveals a novel mechanism: USP44 regulates LUAD’s DDP resistance and malignancy by stabilizing MAOB, offering a new perspective on chemoresistance regulation.

In the core target screening stage, this study integrated DDP resistance–related targets from GSE157692 and GeneCards, identifying core DDP resistance candidates via intersection analysis. Subsequent functional enrichment analysis clarified their biological relevance. KEGG analysis showed significant enrichment in “drug metabolism‐cytochrome P450” and “platinum drug resistance” pathways, consistent with mechanisms reported in previous gastric and ovarian cancer studies such as abnormal metabolic enzyme regulation of drug metabolism and enhanced drug efflux mediating resistance [10–12]. This confirms the critical role of drug metabolic reprogramming in LUAD DDP resistance. GO analysis indicated enrichment in functional modules like “epithelial to mesenchymal transition” and “extracellular space.” Since EMT is a well‐established driver of tumor metastasis and chemoresistance and cytochrome P450‐mediated metabolic imbalance is a known inducer of chemoresistance [13–15], these collectively validate the scientific rigor and rationality of the core candidate screening.

Analysis of genes enriched in KEGG pathways revealed that MAOB was lowly expressed in A549/DDP cells. Previous studies on MAOB have mostly focused on its central neuroprotective effects [16, 17], while its function in tumors has been rarely reported. This work illustrated that MAOB was markedly downregulated in DDP‐resistant cells and LUAD tissues, and its low expression correlated with poor prognosis in patients. This is consistent with the phenomenon observed by Qi et al. [18] in head and neck squamous cell carcinoma: Their study confirmed that MAOB was reduced in HNSCC, and MAOB overexpression inhibited the malignancy of HNSCC cells. Notably, MAOB overexpression in this study effectively reversed DDP resistance, as evidenced by increased cellular sensitivity to the drug, enhanced proliferation inhibition, and promoted apoptosis. In addition, MAOB overexpression also inhibited the migration, invasion, and EMT process of drug‐resistant cells, which aligns with the classical understanding of EMT regulation in LUAD. As a core driver of tumor metastasis and drug resistance, EMT can enhance the invasive ability of cells by endowing them with a mesenchymal phenotype, while reducing their sensitivity to chemotherapeutic drugs; this phenomenon has been confirmed in various solid tumors [19, 20].

To elucidate the upstream regulatory mechanism of MAOB, this study confirmed via bioinformatics prediction and experimental validation that USP44 stabilizes the MAOB protein through deubiquitination modification. USP44, a member of the DUB family, plays a central role in maintaining protein stability by removing Ub chains from substrate proteins, and its functions have been validated in various cancers [21, 22]. For instance, in oral squamous cell carcinoma, USP44 suppresses tumor proliferation and metastasis by stabilizing HEXIM1 [23]; in lung cancer, it restrains cell growth by downregulating the AKT pathway [24]. Notably, in the context of chemoresistance, USP44 overexpression enhances DDP sensitivity in neuroblastoma [25] and overcomes gemcitabine resistance in pancreatic cancer [26]. These studies underscore that USP44 exhibits substrate‐ and context‐dependent roles in tumor biology and treatment response. Earlier research on USP44 has largely centered on its functions in cell cycle control and DNA damage repair [27], whereas recent research has started to uncover its regulatory potential in chemoresistance. Xiao et al. [28] reported that in gastric cancer, USP44 stabilizes ITGB4 via deubiquitination, thereby alleviating DDP resistance, whereas Wu et al. [29] found that USP44 promotes chemoresistance in triple‐negative breast cancer by stabilizing EZH2 protein, suggesting that USP44 exhibits significant substrate‐dependent differences in its regulatory effects on chemoresistance across different cancers. This study further expands this understanding: In LUAD, USP44 exhibits reduced expression in both DDP‐resistant cells and clinical tumor tissues, with its low levels notably linked to unfavorable patient outcomes. At the functional level, USP44 overexpression prolongs the half‐life of MAOB protein via deubiquitination, thereby inhibiting colony formation and suppressing the EMT process. In contrast, MAOB knockdown completely reverses the aforementioned effects of USP44, confirming that MAOB is the key functional effector molecule through which USP44 regulates LUAD chemoresistance and malignant phenotypes. This finding directly links DUB‐mediated regulation of protein stability to DDP resistance in LUAD, further expanding the cognitive boundary of “chemoresistance caused by ubiquitin‐proteasome system (UPS) dysregulation.”

In conclusion, this study elucidates that low MAOB expression mediates DDP resistance in LUAD by inhibiting apoptosis and promoting EMT and identifies USP44‐mediated MAOB stabilization via deubiquitination as the key upstream regulator. The discovery of the USP44‐MAOB axis not only deepens understanding of the molecular network behind LUAD chemoresistance but also provides an experimental basis for developing resistance reversal strategies targeting this axis. However, this study is primarily confined to in vitro cell models. The lack of in vivo validation in animal models is a major limitation, constraining the direct assessment of the therapeutic potential of targeting this axis within a living system. Future research urgently requires validation of the impact of the USP44‐MAOB axis on DDP efficacy in animal models, such as tumor‐bearing mice, and exploration of its correlation with chemotherapy response in clinical samples. Furthermore, the crosstalk between MAOB and other chemoresistance pathways remains to be explored. Addressing these issues will provide more precise theoretical support for the personalized treatment of LUAD.

## Funding

This work was supported by Zhejiang Province Traditional Chinese Medicine Science and Technology Project (Grant No. 2025ZL160).

## Conflicts of Interest

The authors declare no conflicts of interest.

## Supporting Information

Additional supporting information can be found online in the Supporting Information section.

## Supporting information


**Supporting Information 1** Figure S1: MAOB mRNA expression is significantly downregulated in DDP‐resistant LUAD tissues. Relative MAOB mRNA expression was detected by qRT‐PCR in 19 DDP‐sensitive and 27 DDP‐resistant LUAD tissue samples. Data are presented as individual data points with mean ± SD. ∗∗∗*p* < 0.001.

**Supporting Information 2** Figure S2: MAOB knockdown does not affect USP44 protein expression in DDP‐resistant LUAD cells. (A, B) Western blot analysis of MAOB and USP44 protein levels in A549/DDP and PC9/DDP cells transfected with siNC or siMAOB. Data are presented as mean ± SD from three independent biological replicates.  ^∗∗^ *p* < 0.01.

## Data Availability

The data that support the findings of this study are available from the corresponding author upon reasonable request.
