# Supplementary figures and images for: USP44 Stabilizes MAOB via Deubiquitination to Inhibit Cisplatin Resistance in Lung Adenocarcinoma
Source: Int J Genomics. 2026 May 22;2026:7433804. doi: 10.1155/ijog/7433804 (PMC13195626; doi:10.1155/ijog/7433804)

**Relative MAOB  
mRNA expression**

**\*\*\***

**DDP-sensitive**

**DDP-resistant**

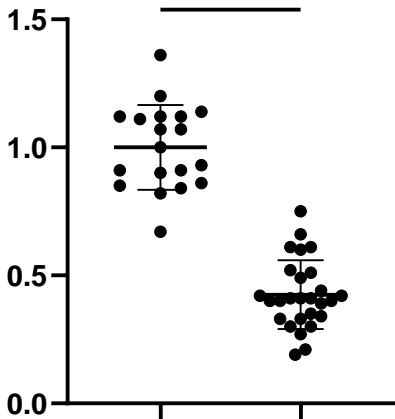

Supplement: Supplementary file 1 — Supporting Information 1 Figure S1: MAOB mRNA expression is significantly downregulated in DDP‐resistant LUAD tissues. Relative MAOB mRNA expression was detected by qRT‐PCR in 19 DDP‐sensitive and 27 DDP‐resistant LUAD tissue samples. Data are presented as individual data points with mean ± SD. ∗∗∗p < 0.001. [file IJOG-2026-7433804-s001.pdf]

**A**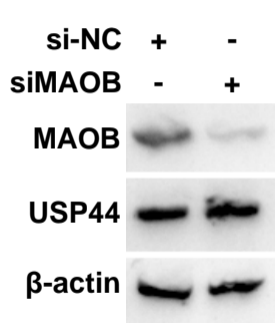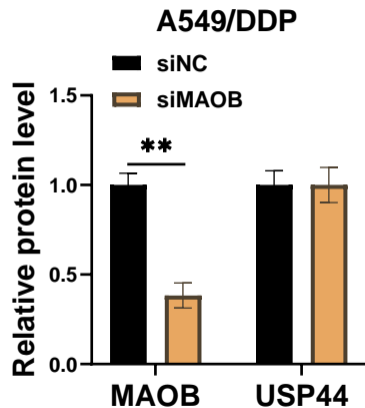**B**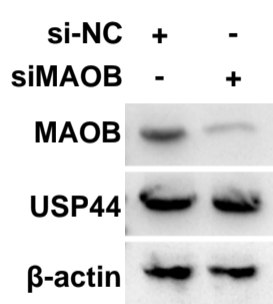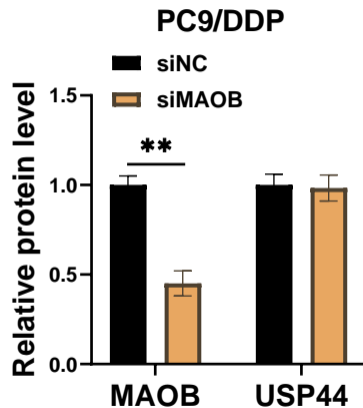

Supplement: Supplementary file 2 — Supporting Information 2 Figure S2: MAOB knockdown does not affect USP44 protein expression in DDP‐resistant LUAD cells. (A, B) Western blot analysis of MAOB and USP44 protein levels in A549/DDP and PC9/DDP cells transfected with siNC or siMAOB. Data are presented as mean ± SD from three independent biological replicates. ∗∗ p < 0.01. [file IJOG-2026-7433804-s002.pdf]
